# Insight into the lncRNA–mRNA Co-Expression Profile and ceRNA Network in Lipopolysaccharide-Induced Acute Lung Injury

**DOI:** 10.3390/cimb45070389

**Published:** 2023-07-24

**Authors:** Yue Shen, Linjing Gong, Fan Xu, Sijiao Wang, Hanhan Liu, Yali Wang, Lijuan Hu, Lei Zhu

**Affiliations:** 1 Department of Pulmonary Medicine, Zhongshan Hospital, Fudan University, Shanghai 200032, China; 15802109693@139.com (Y.S.); 20111210046@fudan.edu.cn (F.X.); wsj_0326@163.com (S.W.); lhhssym@163.com (H.L.); 19111210008@fudan.edu.cn (Y.W.); hu.lijuan@zs-hospital.sh.cn (L.H.); 2 Department of Respiratory and Critical Care Medicine, West China Hospital, Sichuan University, Chengdu 610041, China; gonglinjing@yeah.net; 3 Department of Pulmonary and Critical Care Medicine, Huadong Hospital, Fudan University, Shanghai 200040, China

**Keywords:** acute lung injury, long noncoding RNAs, high-throughput RNA sequencing, expression profile, ceRNA regulatory network, inflammatory response

## Abstract

Long non-coding RNAs (lncRNAs) participate in acute lung injury (ALI). However, their latent biological function and molecular mechanism have not been fully understood. In the present study, the global expression profiles of lncRNAs and mRNAs between the control and lipopolysaccharide (LPS)-stimulated groups of human normal lung epithelial cells (BEAS-2B) were determined using high-throughput sequencing. Overall, a total of 433 lncRNAs and 183 mRNAs were differentially expressed. A lncRNA–mRNA co-expression network was established, and then the top 10 lncRNAs were screened using topological methods. *Gene Ontology* and *Kyoto Encyclopedia of Genes and Genomes* analysis results showed that the key lncRNAs targeting mRNAs were mostly enriched in the inflammatory-related biological processes. Gene set variation analysis and Pearson’s correlation coefficients confirmed the close correlation for the top 10 lncRNAs with inflammatory responses. A protein–protein interaction network analysis was conducted based on the key lncRNAs targeting mRNAs, where IL-1β, IL-6, and CXCL8 were regarded as the hub genes. A competing endogenous RNA (ceRNA) modulatory network was created with five lncRNAs, thirteen microRNAs, and twelve mRNAs. Finally, real-time quantitative reverse transcription-polymerase chain reaction was employed to verify the expression levels of several key lncRNAs in BEAS-2B cells and human serum samples.

## 1. Introduction

Acute lung injury (ALI) and its progression phase of acute respiratory distress syndrome (ARDS) are clinical syndromes with manifestations of progressive dyspnea and refractory hypoxemia caused by intrapulmonary and/or extrapulmonary elements [1]. For decades, ALI and ARDS have been concerning due to their high morbidity and mortality rates. Giacomo et al., have carried out a thorough inquiry into the epidemiology of ARDS patients admitted to the participating intensive care units across 50 countries and found that the period prevalence for ARDS was 10.4%, among which mild, moderate, and severe ARDS patients accounted for 30.0%, 46.6%, and 23.4% of the cohort, respectively [2]. In addition, authoritative clinical research has drawn consistent conclusions that in-hospital mortality of ARDS patients exceeded 30% [3]. Despite active clinical management, such as the application of mechanical ventilation, prone positioning, neuromuscular blockade, and pharmacotherapy, there seems to be no discernible impact [4].

It has been extensively recognized that uncontrolled and excessively amplifying inflammatory response is the core pathogenesis in the acute phase of ALI/ARDS. Carolyn et al., were the first to identify two subphenotypes of ARDS. Compared to subphenotype 1, subjects in the subphenotype 2 (hyperinflammatory subphenotype) group showed similar clinical signatures, with higher inflammatory biomarker levels in the plasma, higher prevalence of sepsis, and higher mortality rates [5]. Another study has also reported that these subphenotypes responded diversely to a fluid management strategy, one which could be classified using a three-variable model of interleukin-8 (IL-8), bicarbonate, and tumor necrosis factor receptor-1 (TNFr-1) [6]. Nevertheless, the majority of researchers have mainly focused on the mechanisms and functions of inflammation-related protein-coding genes in ALI/ARDS. However, the beginning of the postgenomic epoch has revealed a brand-new field of regulatory elements in the human genome [7]. Once regarded as junk DNA, tens of thousands of lncRNAs have been discovered due to the rapid advances in high-throughput sequencing. Accumulating evidence has suggested that lncRNAs may play crucial roles in different pathophysiological processes, such as proliferation, migration, apoptosis, and mitophagy, and possibly regulate genes epigenetically, transcriptionally, and post-transcriptionally [8,9,10,11]. In recent years, several lncRNAs have been shown to participate in the process of acute lung inflammation through diverse patterns [12]. Hu et al., have discovered that lncRNA SNHG1, acting as a proinflammatory driver, physically interacted with HMGB1 in ALI, leading to cytokine storms [13]. Moreover, prior clinical research has indicated that the serum level of lncRNA GAS5 was lower in the COVID-19 patients compared to the control group [14]. Therefore, lncRNAs have been shown to be potentially promising diagnostic biomarkers in ALI/ARDS. Further screening of serum biological indicators with predictive value is of great significance for targeted therapy.

The present study aimed to explore the expression profile relationship between mRNAs and lncRNAs in LPS-treated BEAS-2B cells and human serum samples, thus identifying lncRNAs that participated in the pathophysiology process of ALI using bioinformatics analysis and biological experiments in vitro and in vivo.

## 2. Materials and Methods

### 2.1. Clinical Data Collection and Sample Preparation

The present study was performed in accordance with the Declaration of Helsinki and was approved by the ethics committee of Zhongshan Hospital, which is affiliated with Fudan University (approval no. B2021-183(2)). All participants signed the written informed consent form before recruitment.

A total of six patients with ALI/ARDS and six healthy participants were recruited at Zhongshan Hospital, Fudan University. The inclusion criterion was an ALI/ARDS diagnosis according to the Berlin consensus definition (2012) [1]. The exclusion criteria were: age of <18 or >80 years, pregnancy, immunosuppression treatment, terminal stage of malignant tumor disease, and enrollment in other experimental treatment protocols. Peripheral blood samples were collected from healthy participants and ALI/ARDS patients using ethylenediaminetetraacetic acid tubes. For serum preparation, blood samples were centrifuged at 3000× *g* at 4 °C for 10 min. The supernatants were then removed, re-centrifuged at 13,000 rpm at 4 °C for 10 min, and stored at −80 °C until further use.

### 2.2. Cell Culture and Treatment

Human normal lung epithelial cells (BEAS-2B) were purchased from the Chinese Academy of Sciences Cell Bank (Shanghai, China) and cultivated in Dulbecco’s modified Eagle’s medium (#D6429, Sigma Aldrich, Saint Louis, MO, USA) with 10% fetal bovine serum (#10270-106, Gibco, São Paulo, Brazil) and 1% penicillin/streptomycin (#V900929, Sigma Aldrich, Saint Louis, MO, USA) at 37°C in a 5% CO_2_ incubator. BEAS-2B cells were treated with LPS (*Escherichia coli* (O111:B4), #L2630, Sigma-Aldrich, Saint Louis, MO, USA) at concentrations of 0, 5, 10, 25, and 50 µg/mL for 24 h for investigation, and 10 µg/mL was finally chosen for ALI establishment. The cells were randomly divided into the lipopolysaccharide (LPS) and control (without LPS stimulation) groups. Each group contained three samples.

### 2.3. EDU Detection

Cell proliferation was measured using a BeyoClick™ EDU kit with Alexa Fluor 488 (#C0071S, Beyotime, Shanghai, China). BEAS-2B cells were seeded into a six-well plate overnight. After being stimulated by LPS at a concentration of 10 µg/mL for 24 h, the cells were cultured in EDU dyeing solution for 2 h at a final concentration of 10 µM. After using the BeyoClick EDU Cell Proliferation Kit with Alexa Fluor 488, Hoechst 33342 was utilized for nuclear staining according to the manufacturer's instructions. Images were captured using a microscope (Olympus IX51, Tokyo, Japan).

### 2.4. Cell Viability Assay

Cell viability was detected using a Cell Counting Kit-8 (CCK-8) assay (#KGA317, KeyGEN BioTECH, Jiangsu, China). BEAS-2B cells were seeded into 96-well plates and treated with LPS at doses of 0, 5, 10, 25, and 50 µg/mL for 24 h. Then, the CCK-8 reagent was added into every well at a concentration of 10 µL per 100 µL of medium and incubated for 2 h at 37 °C, protected from light. The absorbance was measured at 450 nm using a microplate spectrophotometer (Thermo Fisher Scientific, Waltham, MA, USA).

### 2.5. Reactive Oxygen Species (ROS) Detection

BEAS-2B cells were collected to quantify intracellular ROS using dichloro-dihydro-fluorescein diacetate (DCFH-DA) fluorescent probes (#S0033S, Beyotime, Shanghai, China). After the treatment with 10 µg/mL LPS for 24 h, the cells were stained with a DCFH-DA probe for 20 min in a 37 °C incubator at a dose of 10 µM, diluted with serum-free DMEM, and washed three times with serum-free DMEM. Finally, the ROS activity was captured using FACSCalibur (BD Biosciences, San Jose, CA, USA). Quantification was conducted using FlowJo software (Tree Star, San Carlos, CA, USA).

### 2.6. RNA Isolation and Library Preparation

Total RNA samples from BEAS-2B cells and human serum samples were extracted using a TRIzol reagent (#15596018, Invitrogen, Carlsbad, CA, USA). RNA purity and quantification data were acquired using absorbances of 260/280 and 260/230 ratios using a Nanodrop 2000 spectrophotometer (Thermo Fisher Scientific, Waltham, MA, USA). Agilent 2100 Bioanalyzer (Agilent Technologies, Santa Clara, CA, USA) was used when evaluating the cells in order to assess the integrity of the isolated RNA. Finally, libraries were constructed employing TruSeq Stranded Total RNA with Ribo-Zero Gold (Illumina, San Diego, CA, USA, Cat. No. RS-122-2301) according to the manufacturer’s protocols.

### 2.7. RNA-Seq Analysis

The libraries were sequenced on an Illumina Novaseq 6000 platform, and 150-bp paired-end reads were generated. Raw data in fastq format were first processed using the Trimmomatic software [15]. Briefly, clean data were obtained from raw data by removing reads containing an adapter and ploy-N or those of low quality. Sequencing reads were mapped to the human genome (GRCh38) using HISAT2 [16]. For mRNAs and lncRNAs, differential expression analysis was conducted using the DESeq (2012) R package [17], where *p*-value of <0.05 and |log_2_(fold change)| >0.58 were considered to be the thresholds for a significant difference. Differentially expressed lncRNAs and mRNAs were called DElncRNAs and DEmRNAs, respectively. For lncRNAs, the transcriptome from each dataset was assembled independently using the Cufflinks 2.0 program [18]. All transcriptomes were pooled and merged to generate a final transcriptome using Cuffmerge (Cufflinks 2.0). The characteristics (including length, type, and number of exons) of lncRNA were analyzed after screening. Then, the novel predicted lncRNAs and known lncRNAs (from the NCBI and *Ensemble* databases) were both used for expression calculation and differential screening.

### 2.8. The lncRNA–mRNA Co-Expression Network

The lncRNA–mRNA co-expression network, also known as the coding–noncoding (CNC) co-expression network, was constructed by calculating Pearson’s correlation coefficients among DElncRNAs and DEmRNAs (*p* < 0.05, |r| ≥ 0.8). The final selected CNC network was constructed using the Cytoscape software 3.9.0. 

### 2.9. Cis- and Trans-Regulated Gene Analysis

*Cis*-acting lncRNAs are more likely to regulate the nearby genes with strong co-expression patterns. Based on the co-expression network results, feelnc [19] software was used to search all coding genes within 100 kbp upstream or downstream of DElncRNAs. The significantly co-expressed (*p* < 0.05, |r| ≥ 0.8) pairs were identified. According to the condition that co-expressed lncRNA and mRNA can bind directly through at least 10 bases and that the binding free energy is not greater than −100, *trans*-regulated mRNAs were regulated using RNA interaction software research-2.0. The *trans*-regulating lncRNA–mRNA network was established using the Network [20] software package.

### 2.10. Gene Functional Enrichment Analysis

To explore the role of DElncRNAs, *Gene Ontology* (GO) and the *Kyoto Encyclopedia of Genes and Genomes* (KEGG) analyses of mRNAs in the CNC network were performed via DAVID (http://www.david.ncifcrf.gov accessed on 20 June 2022) [21]. The GO enrichment contained biological processes (BPs), molecular functions (MFs), and cellular components (CCs). The KEGG analysis reflected the most important signaling pathways. A *p*-value of <0.05 was considered to indicate a statistically significant difference.

### 2.11. Gene Set Variation Analysis (GSVA)

The gene sets relating to the immune and inflammation processes were obtained from the GSEA (http://www.gsea-msigdb.org accessed on 20 June 2022). The enrichment score for each sample was evaluated using the “GSVA” R package under default parameters. Then, the heat map of GSVA results, as well as the expression of lncRNAs, were presented via the “pheatmap” R package. The relationship between the expression of top lncRNAs and the enrichment score of the gene sets was determined using Spearman’s correlation analysis.

### 2.12. Construction of Protein–Protein Interaction (PPI) Network

The PPI network for key lncRNAs targeting mRNAs was established using STRING (version 11.5, http://www.string-db.org accessed on 15 June 2022) and then visualized with Cytoscape (version 3.9.0). The MCODE algorithm was used to extract the core components from the original network, which shared high topological parameter scores such as Degree.

### 2.13. LncRNA–microRNA–mRNA ceRNA Network Construction

**Figure 1:**
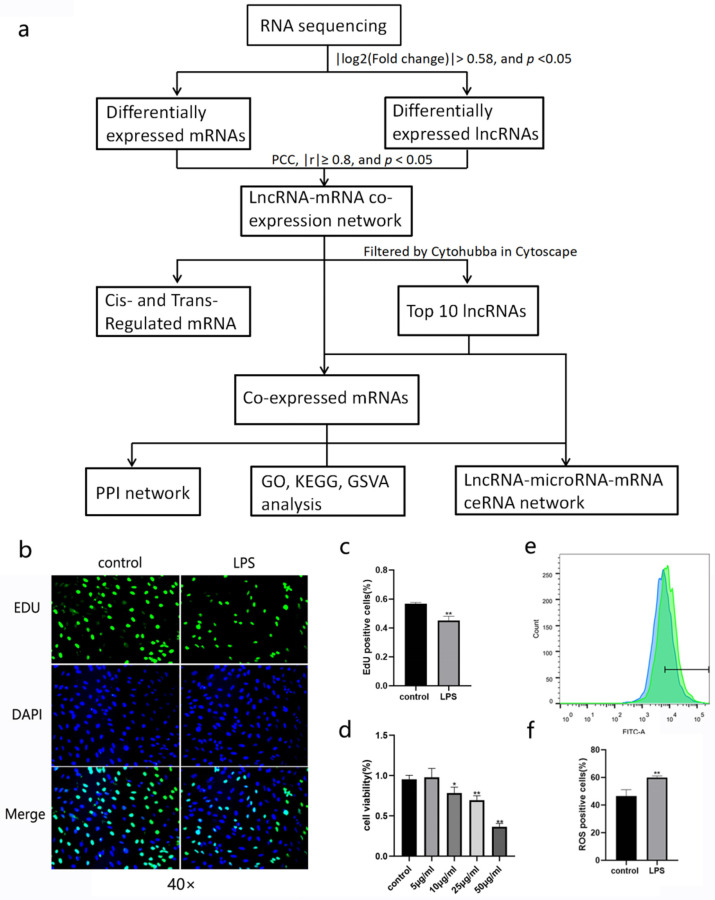
Workflow and ALI model establishment of this study. (**a**) Flowchart of this work. (**b**,**c**) EDU assay for cell proliferation detection, (**d**) cell viability calculation, and (**e**,**f**) ROS production in LPS-stimulated BEAS-2B cells (green portion) and control group (blue portion). Values are the mean ± SD; * *p* < 0.05 vs. the control group, ** *p* < 0.01 vs. the control group.

The “multiMiR” R package (version 2.3.0) [22] was used to predict microRNAs that would target DEmRNAs. Generally, the “multiMiR” R package consists of eight predicted miRNA–target interaction databases: *DIANA-microT-CDS*, *ElMMo*, *MicroCosm*, *miRanda*, *miRDB*, *PicTar*, *PITA*, and *TargetScan*. In the present study, microRNA–mRNA pairs had binding sites predicted by at least three databases simultaneously. Furthermore, lncRNA–microRNA interactions were predicted using the *miRDB* database. Top 10 ranked microRNAs for each key lncRNA in the CNC network were selected. Finally, all filtered miRNA–mRNA and lncRNA–miRNA pairs were integrated into the lncRNA–microRNA–mRNA competing endogenous RNA (ceRNA) network. The workflow of this study is presented in Figure 1a.

### 2.14. Expression Profile Validation Using RT-qPCR

After extraction and qualification, the RNA was reverse transcribed into cDNA using PrimeScript™ RT reagent Kit (#RR037A, Takara, Japan). RT-qPCR was performed using TB Green® Premix Ex Taq™ II (#RR820A, Takara, Japan) on a Quant Studio 5 system. Primers are represented in Supplementary Table S1.

### 2.15. Statistical Analysis

Data were presented as mean ± standard deviation (SD) or the number of cases (%). Difference between two independent groups was analyzed using an unpaired Student’s *t*-test. The baseline characteristics were compared with Fisher’s exact test. A *p*-value < 0.05 was considered to be a statistically significant difference. All statistical analyses were conducted using GraphPad Prism 8.0 (GraphPad, La Jolla, CA, USA).

## 3. Results

### 3.1. Establishment of ALI Model in BEAS-2B Cells

BEAS-2B cells were severally treated with LPS at concentrations of 1, 5, 10, 25, and 50 µg/mL for 24 h. Then, CCK-8 assay results showed that the viability of BEAS-2B cells decreased in a dose-dependent manner (Figure 1d). When the concentration of LPS increased to a 10 µg/mL or higher level, the viability of BEAS-2B cells was significantly lower than that of the control group. EDU detection also demonstrated that 10 µg/mL LPS impaired cell proliferation (Figure 1b,c). According to the fluorescence results, 10 µg/mL LPS also triggered an increase in ROS (Figure 1e,f). Therefore, we chose 10 µg/mL LPS for ALI model establishment in BEAS-2B cells.

### 3.2. Characterization of lncRNAs and mRNAs

About 98,775,859–107,437,097 raw reads for each sample were generated, and about 97,622,425–106,094,920 clean reads were retained for subsequent analyses. There was no remarkable difference in FPKM value distribution and gene expression density between the ALI and control groups, either for mRNA or lncRNA (Supplementary Figure S1a–d). Generally, the FPKM values for lncRNA were at a lower level than those for mRNA, and the majority of them were concentrated in the 0–0.5 range. The Coding Potential Calculator (CPC) [23], the predictor of long non-coding RNAs and messenger RNAs based on an improved k-mer scheme (PLEK) [24], the Coding-Non-Coding Index (CNCI) [25], and a database of protein families (Pfam) [26] were four tools used to assess the protein-coding potential of a transcript and distinguish lncRNAs from mRNAs via different algorithms. The intersection of those four tools was exploited to predict 1418 novel lncRNAs (Supplementary Figure S1e). Then, the novel predicted lncRNAs and annotated lncRNAs (from the NCBI and *Ensemble* databases) were divided into two categories: 5975 sense lncRNAs and 12,387 antisense lncRNAs. Supplementary Figure S1f–g shows the details of this classification based on the genomic location. The characteristics of lncRNAs, including length, number of exons, and distribution on the chromosome, were also represented. The lncRNAs that were >2000 nt in length accounted for the majority of lncRNAs, and their average length was nearly 2052 nt (Supplementary Figure S1h). Most of the lncRNAs contained two to five exons, and chromosome 1 had the largest number of lncRNAs among all chromosomes (Supplementary Figure S1i–j).

### 3.3. Expression Profiles of lncRNAs and mRNAs 

**Figure 2:**
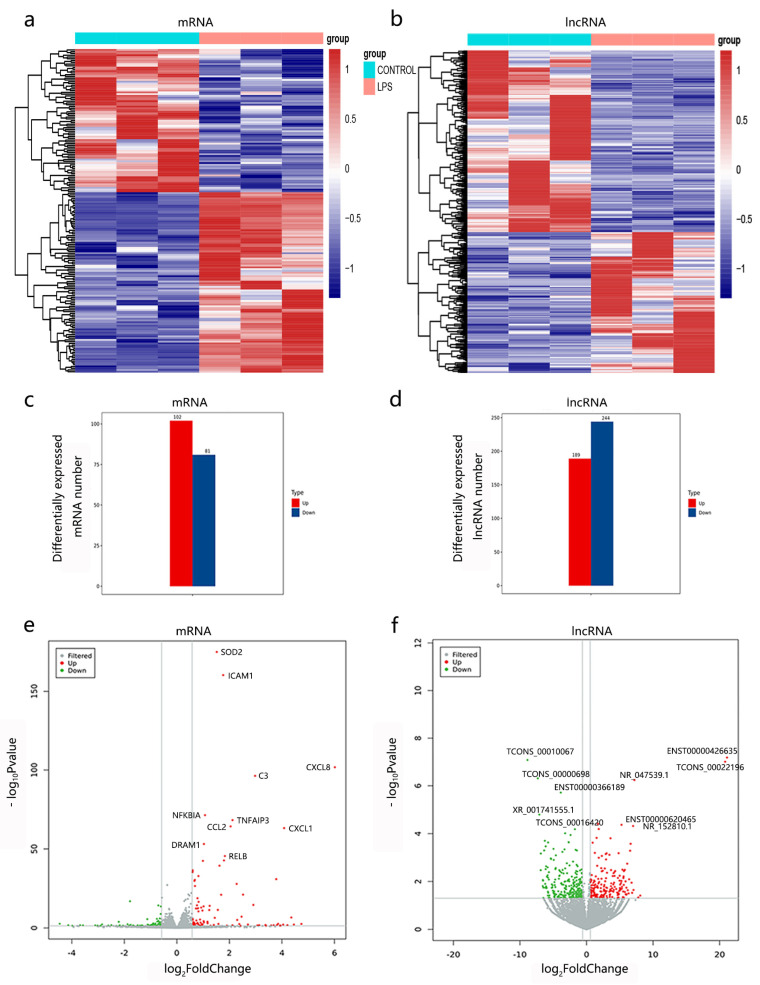
The mRNA and lncRNA expression profiles in LPS-induced BEAS-2B cells. (**a**,**b**) The heatmap of differentially expressed mRNAs (DEmRNAs) and lncRNAs (DElncRNAs). Three LPS-induced samples and three control samples are incorporated; each column represents a sample and each row represents a transcript. (**c**,**d**) Numbers of DEmRNAs and DElncRNAs. (**e**,**f**) The volcano plots of DEmRNAs and DElncRNAs. The filtered transcripts with no significance are in grey, upregulated transcripts are in red, and downregulated transcripts are in green. The top 10 significantly changed mRNAs and lncRNAs are labeled in volcano plots.

Compared to the control group, a total of 183 DEmRNAs were uncovered, including 102 upregulated mRNAs and 81 downregulated mRNAs (*p* < 0.05, |log_2_(fold change)| > 0.58) (Figure 2a,c). In addition, 433 DElncRNA transcripts were identified in the LPS-induced ALI model in BEAS-2B cells, among which 189 lncRNAs were upregulated and 244 lncRNAs were downregulated (*p* < 0.05, |log_2_(fold change)| > 0.58) (Figure 2b,d). The expression profiles of the lncRNA are presented in Supplementary Table S2. The top 10 significantly changed lncRNAs and mRNAs are shown in Figure 2e,f.

### 3.4. Co-Expression Network of lncRNA–mRNA

**Figure 3:**
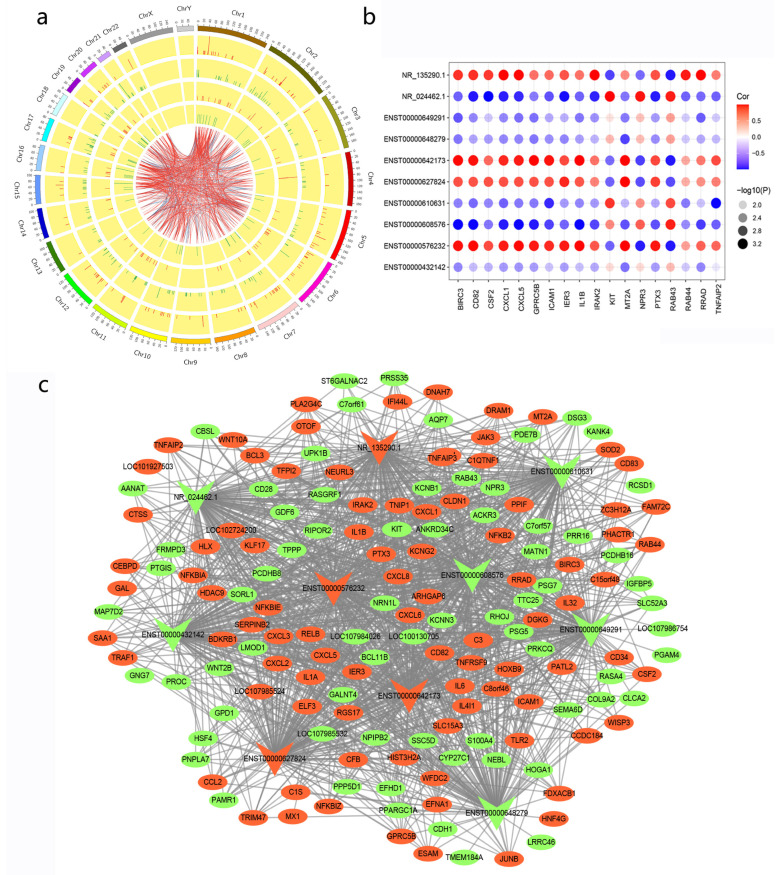
Correlation of DElncRNAs and DEmRNAs. (**a**) Co-expression Circos map of the original CNC network. The outer layer is the distribution diagram of chromosomes. The second and third layers represent DEmRNAs displayed on chromosomes. The red line indicates up-regulation while the green indicates down-regulation, and a higher column implies more genes in this interval. The innermost two layers were the distribution of DElncRNAs on chromosomes. (**b**) Correlation of the top 10 lncRNAs with highly correlated mRNAs (*p* < 0.05, |r| > 0.99). (**c**) Co-expression network of the top 10 lncRNAs and their correlated mRNAs. The red color represents up-regulation and green represents down-regulation, while the circle shape represents mRNAs and the triangle represents lncRNAs.

To explore the relationship between DElncRNAs and DEmRNAs, an original CNC network analysis was conducted. Pearson’s correlation coefficients (*p* < 0.05, |r| ≥ 0.8) were calculated to screen the differentially expressed lncRNA–mRNA pairs in the original CNC network. The distribution of those lncRNAs and mRNAs on chromosomes is shown in Figure 3a. A cytohubba algorithm was used to filter the top 10 lncRNAs that had a strong correlation with DEmRNAs and to comprehensively sort them using several topological analysis methods, including Degree, Edge Percolated Component (EPC), Closeness, and Radiality (Supplementary Table S3). Among the top 10 lncRNAs mentioned above, there are four upregulated lncRNAs and six downregulated lncRNAs (Table 1). Several lncRNA–mRNA pairs with a relatively high correlation (*p* < 0.05, |r| > 0.99) were chosen to build a dotplot, one which explains the relationship among the top lncRNAs and their highly correlated mRNAs more vividly (Figure 3b). The final CNC network consisted of 10 lncRNAs and 161 mRNAs (Figure 3c).

### 3.5. Cis- and Trans-Regulated Gene Analysis

**Figure 4:**
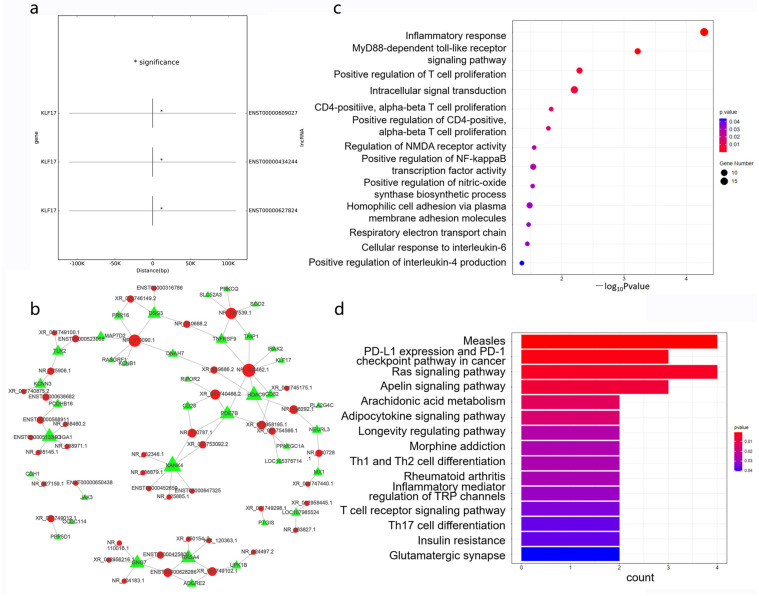
Functional enrichment of cis- and trans-regulated genes of DElncRNAs in the original CNC network. (**a**) The result of cis-regulation. The left and right of the *Y* axis are mRNA and lncRNA, respectively, and the *X*-axis is the distance between mRNA and lncRNA. A negative value indicates upstream and a positive value indicates downstream. (**b**) The result of trans-regulation. The green triangle represents mRNAs, and the red circle represents lncRNAs. (**c**) GO analysis of cis- and trans-regulated genes. (**d**) KEGG pathway analysis of cis- and trans-regulated genes. * *p* < 0.05.

It is widely accepted that lncRNAs are roughly divided into two types: those that regulate neighboring gene expression and/or chromatin states in cis, and those that leave the transcription site and perform functions in trans [27]. Only KLF17 has been predicted to be a cis-regulated gene. Its correlated lncRNAs were ENST00000609027, ENST00000434244, and ENST00000647824 (Figure 4a). A large number of trans-regulated mRNAs have been predicted (Figure 4b). To a certain degree, the potential functions of cis- and trans-regulated genes targeted by DElncRNAs possibly reflected the roles of lncRNAs in response to LPS stimulation. An enrichment analysis of DEmRNAs in the original CNC network was also conducted. GO analysis showed that the inflammatory response, MyD88-dependent toll-like receptor signaling pathway, and positive regulation of T cell proliferation were obviously enriched, and the changes of these three signaling pathways were consistent with previous studies [28] (Figure 4c). Intriguingly, the top three KEGG terms were measles, PD-L1 expression and PD-1 checkpoint pathway in cancer, and Ras signaling pathway, which was slightly unusual in LPS-induced ALI (Figure 4d).

### 3.6. Enrichment Analysis of Differentially Expressed Genes

**Figure 5:**
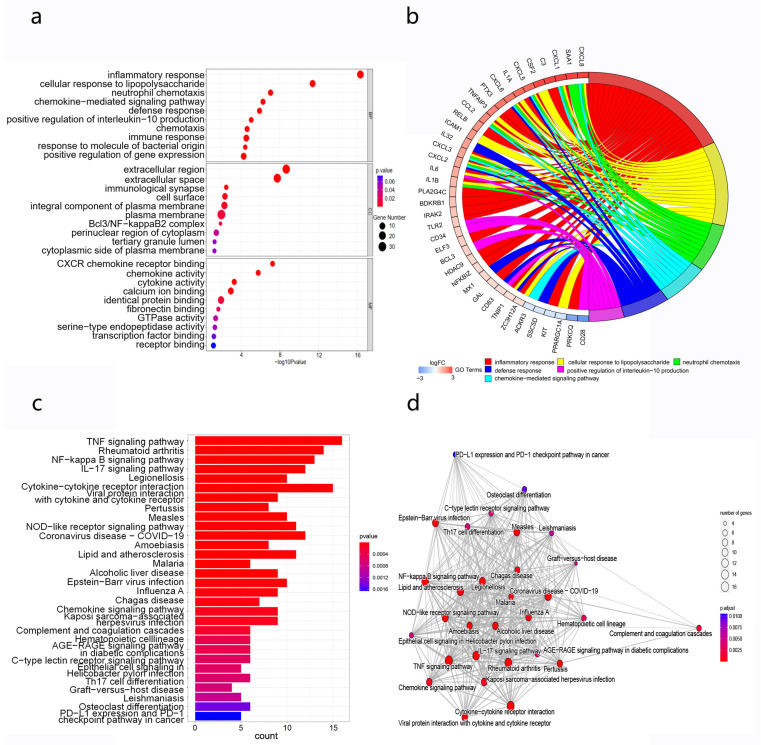
Functional enrichment of DEmRNAs, correlated with the top 10 lncRNAs. The top 10 lncRNAs were selected by several topological parameters, using the CytoHubba algorithm in Cytoscape. (**a**) GO analysis and (**b**) visualization of distribution on DEmRNAs in seven GO-enriched terms. (**c**) KEGG pathway analysis and (**d**) the interaction network for KEGG-enriched pathways. The size of the circle represents gene number in the pathway, and the color of the circle represents adjustive *p*-value.

The latent functions and mechanisms of the top 10 lncRNAs were explored by assessing GO and KEGG enrichment results for DEmRNAs in the lncRNA–mRNA co-expression network. The top three BP terms were “inflammatory response”, “cellular response to lipopolysaccharide”, and “neutrophil chemotaxis”. For CCs, “extracellular region”, “extracellular space”, and “immunological synapse” were the distinctly enriched terms. “CXCR chemokine receptor binding”, “chemokine activity”, and “cytokine activity” were the first three MF enriched terms (Figure 5a,b). The KEGG enrichment analysis results demonstrated that DEmRNAs in the CNC network were significantly involved in the “TNF signaling pathway”, “NF-kappa B signaling pathway”, “IL-17 signaling pathway” and “NOD-like receptor signaling pathway” (Figure 5c,d).

### 3.7. Relationship of Candidate lncRNAs with Immune and Inflammatory Responses

**Figure 6:**
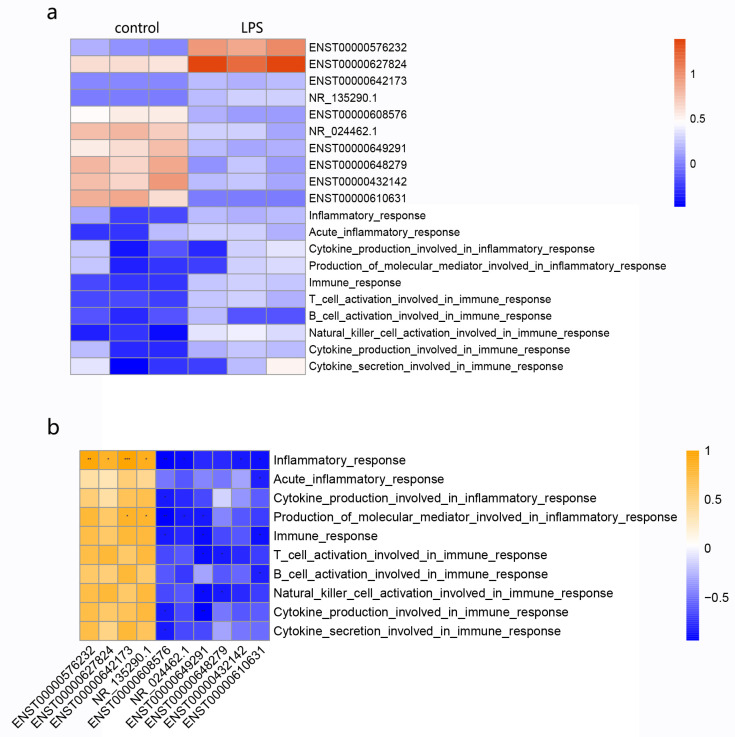
Correlation analysis for the expression of the top 10 lncRNAs with immune and inflammatory function enrichment scores. (**a**) The first heatmap shows the expression of the top 10 lncRNAs and the enrichment scores of immune and inflammatory functions in each sample calculated by GSVA. (**b**) The second heatmap presents a correlation of the top 10 lncRNAs with different immune and inflammatory responses. * *p* < 0.05 vs. the control group, ** *p* < 0.01 vs. the control group, *** *p* < 0.001 vs. the control group.

According to the GO and KEGG results, the production and activation of cytokines, chemokines, and lymphocytes played vital roles during the development of ALI. Therefore, the uniformity of lncRNA alterations and immune/inflammation signatures was investigated. GSVA was carried out to calculate the enrichment scores for each sample. The heatmap of GSVA results, together with the expression of each lncRNA, is shown in Figure 6a. Correlation analysis indicated that the expression of upregulated lncRNAs, such as ENST00000576232, ENST00000627824, ENST00000642173, and NR_135290.1, presented highly positive correlations with inflammatory responses (*p* < 0.05, |r| > 0.8). On the contrary, downregulated lncRNAs also showed negative correlations with inflammatory responses (*p* < 0.05, |r| > 0.8), except for ENST00000649291 and ENST00000648279 (Figure 6b). These results indicated that these key lncRNAs might interact closely with the process of inflammation in LPS-induced ALI.

### 3.8. PPI Analysis

**Figure 7:**
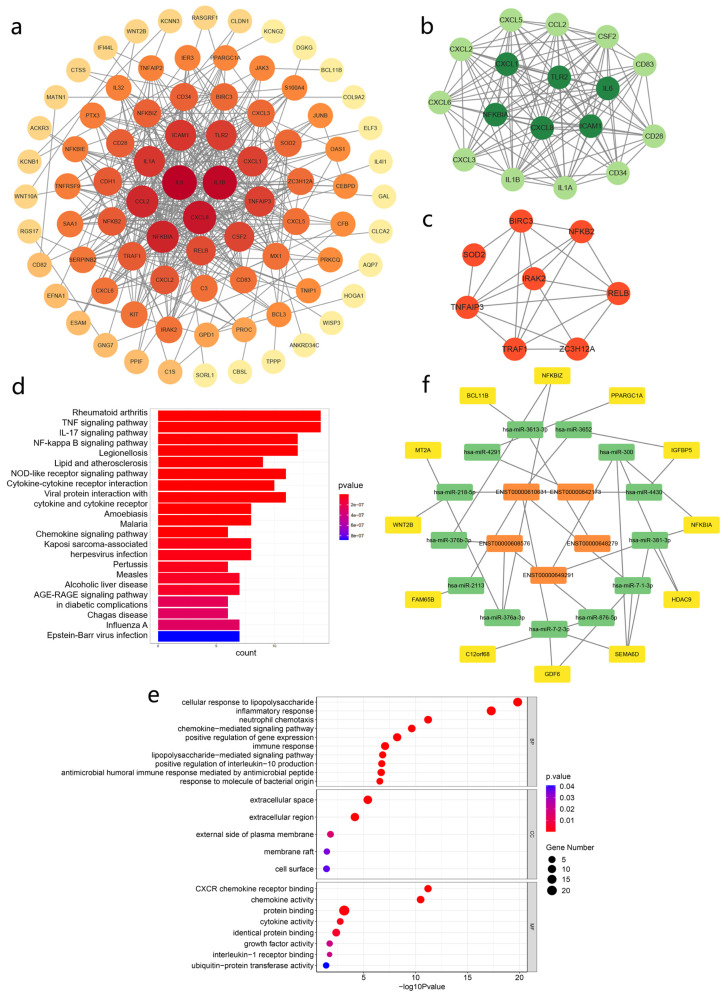
PPI network and ceRNA network. (**a**) PPI network of DEmRNAs correlated with the top 10 lncRNAs. (**b**) Hub gene module 1 in PPI network selected by MCODE. (**c**) Hub gene module 2 in PPI network selected by MCODE. (**d**,**e**) KEGG and GO analysis of all the hub genes from module 1 and module 2. (**f**) LncRNA–miRNA–mRNA ceRNA network; the orange color represents lncRNAs, green represents microRNAs and yellow represents mRNAs.

To investigate the interaction among DEmRNAs in the CNC network and identify the most significant clusters, a PPI network that included 82 nodes and 422 edges was constructed using STRING and visualized with Cytoscape (Figure 7a). IL-1β, IL-6, and CXCL8 were at the central position of the network. In addition, two hub gene modules using the MCODE plug-in were also obtained. Module 1 contained seventeen nodes, including CXCL1, CXCL 2, CXCL 3, CXCL 5, CXCL 6, CXCL 8, CD28, IL-1α, IL-1β, IL-6, CD34, CD83, CCL2, CSF2, ICAM1, NFKBIA, and TLR2. Module 2 comprised eight genes, including SOD2, IRAK2, BIRC3, RELB, TRAF1, NFKB2, TNFAIP3, and ZC3H12A (Figure 7b,c). To further explore the biological functions of these hub genes, GO and KEGG were performed (Figure 7d,e).

### 3.9. Establishment of a lncRNA–microRNA–mRNA ceRNA Regulatory Network

A lncRNA–microRNA–mRNA ceRNA regulatory network was built to further explore the role that top lncRNAs could play in ALI. The ceRNA network was based on the top 10 lnRNAs and 161 mRNAs in the CNC network. The final ceRNA network contained five lncRNAs, thirteen miRNAs, and twelve mRNAs, which reflected the potential RNA cross-talks in the process of ALI (Figure 7f).

### 3.10. Validation of Selected lncRNA Using RT-qPCR

**Figure 8:**
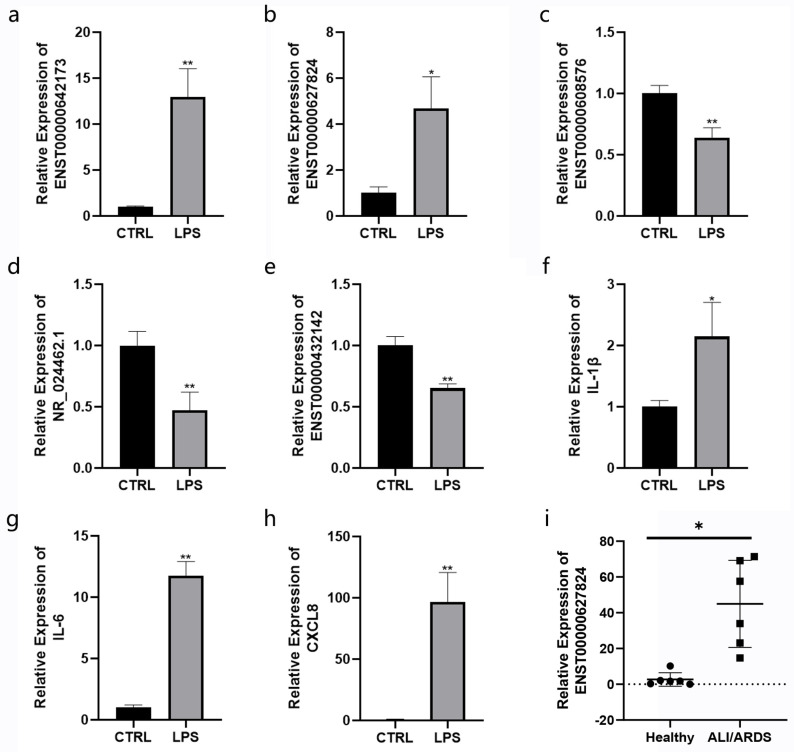
Validation of the expression profiles of key lncRNAs and mRNAs by RT-qPCR. (**a**–**e**) Validation of five randomly selected top lncRNAs and (**f**–**h**) three hub genes in the control group, as well as LPS-induced BEAS-2B cells. (**i**) The expression of ENST00000627824 in human serum samples of healthy participants and ALI/ARDS patients. The round dots represented healthy participants and the square dots represented ALI/ARDS patients. Data are presented as the mean ± SD. * *p* < 0.05 vs. the control group, ** *p* < 0.01 vs. the control group.

RT-qPCR was performed to validate the expression profiles of top lncRNAs. Five lncRNAs were randomly selected, and RT-qPCR validation assay results in BEAS-2B cells implied that the changes in ENST00000642173, ENST00000627824, ENST00000608576, NR_024462.1, and ENST00000432142 were consistent with RNA-seq outcomes (Figure 8a–h) in which ENST00000642173 had the largest amplitude. To search for potential biomarkers in ALI patients, serum samples from six healthy participants and six ALI/ARDS patients (Table 2) were collected to compare their expression levels of ENST00000627824 using RT-qPCR. The results showed that the expression levels of ENST00000627824 in the ALI group were significantly higher than those in the healthy group (*p* < 0.05), which indicated that ENST00000627824 likely played an essential role in the development and progression of ALI (Figure 8i).

## 4. Discussion

ALI and ARDS are clinical syndromes characterized by progressive dyspnea and refractory hypoxemia. They have become concerning due to their high morbidity and mortality [29]. For decades, biomarkers of ALI/ARDS, such as inflammatory cytokines and chemokines, have been continuously studied [30,31,32]. Along with the growing development of high-throughput RNA sequencing, lncRNAs have stood out as an important area in the research of physiological and pathological processes of various diseases [33]. Therefore, it is necessary to elucidate the underlying MFs and BPs of lncRNAs in the development of ALI. The present study investigated the lncRNA–mRNA co-expression profile and carried out bioinformatics analysis of important lncRNAs that might undertake a significant part in the pathophysiological process of ALI. In general, 433 DElncRNAs (189 upregulated, 244 downregulated) and 183 DEmRNAs (102 upregulated, 81 downregulated) were identified using RNA-seq. Then, a CNC network according to Pearson’s correlation coefficients was constructed to identify the top 10 most significant lncRNAs that have a leading role in the occurrence and development of ALI using topological methods. Notably, despite the significance and large-scale change of top10 lncRNAs between the control and LPS group depending on *p*-value, most of the data showed insignificance after FDR correction, which was probably due to large variations between biological replicates. To make the data more trustable, we validated the top 10 lncRNAs on independent biological replicates randomly.

GO enrichment analysis results revealed that the target mRNAs of lncRNAs were mostly involved in the inflammatory responses, cellular response to lipopolysaccharide, immunological synapse, BCL3/NF-kappaB complex, and CXCR chemokine receptor binding. It has been widely accepted that the ALI reaction to severe pulmonary microbial infection originates from the recognized immune pathogens that cause a pro-inflammatory immune response [34]. Liu et al., have reported a lncRNA lnc-Cxcl2 located near chemokine genes in mouse lung epithelial cells, one which impeded increased expression of CXCL2, promoting neutrophil recruitment and other inflammatory responses in the lung after influenza virus infection [35]. 

KEGG analysis revealed that the majority of target DEmRNAs were related to the TNF signaling pathway, NF-kappa B signaling pathway, IL-17 signaling pathway, legionellosis, cytokine–cytokine receptor interaction, pertussis, measles, and NOD-like receptor signaling pathway. It is worth mentioning that the Toll-like receptor signaling pathway (hsa04620), necroptosis (hsa04217), and MAPK signaling pathway (hsa04010) were also enriched (*p* < 0.05; gene numbers ≥ 5), but were not represented on the barplot due to their low ranking. Liang et al. found that lncRNA Malat1 not only activated the NF-kappa B signaling pathway, but also acted as a direct transcriptional target of LPS-induced NF-κB activation [36]. LINC00649 was revealed to recruit TAF15, an element stabilizing the expression of mitogen-activated protein kinase 6 (MAPK6), thus activating the MAPK signaling pathway in lung squamous cell carcinoma [37]. Interestingly, necroptosis is a newly identified form of programmed cell death. It has been suggested that lncRNA TRINGS protected cancer cells from death by inhibiting STRAP-GSK3β-NF-κB necrotic signaling [38]. However, few studies have described the role of lncRNA in necroptosis in the lung tissue, a topic which requires further investigation.

Based on the gene function enrichment analysis results stated above, the top 10 lncRNAs shared a close connection with immunological and inflammatory functions. Hence, GSVA was carried out to calculate the enrichment scores for immune- and inflammation-related gene sets for each sample. The correlation of candidate lncRNAs with those gene sets was then also measured. Interestingly, among the four upregulated lncRNAs, ENST00000642173 ranked as the most significant one, correlating to “Inflammatory response” (*p* = 0.00, r = 1.00), which indicated that it possibly participated deeply in LPS-induced ALI. Generally, downregulated lncRNAs appeared to negatively correlate with immune functions more closely than did upregulated lncRNAs. Overall, the link among the top 10 lncRNAs and inflammatory responses was fully identified using bioinformatics analysis, which required further verification in vivo and in vitro.

From the PPI analysis results, nodes in module 2 seemed to have been less reported on by the ALI studies compared to those in module 1. IRAK2 is a member of the interleukin-1 receptor-associated kinase family. Although a large number of research studies have explored the function of IRAK1 in lung injury [39], the role of IRAK2 still has not been fully identified. Guo et al., have found that IRAK2 knockdown ameliorated LPS-induced inflammation response and miR-497a-5p mitigated LPS-stimulated lung injury by targeting the IRAK2-NF-κB pathway [40]. ZC3H12A (also known as Regnase-1 or MCPIP-1) has rarely been studied in the lung [41]. ZC3H12A deficiency in mouse alveolar macrophages represented severe pulmonary arterial hypertension, while IL-6 and IL-1β may have served as potential targets for ZC3H12A in alveolar macrophages [42]. However, the way ZC3H12A influences the ALI process remains unidentified because there has been no related research published. Compared to module 2, hub genes in module1 were mostly classic inflammatory mediators, whichwas consistent with the GO and KEGG analysis results.

According to the ceRNA regulatory network, ENST00000649291 and ENST00000610631 may function as ceRNAs for upregulated hsa-miR-381-3p and hsa-miR-300, as well as hsa-miR-7-1-3p and hsa-miR-7-2-3p, respectively, thus downregulating SEMA6D. Furthermore, hsa-miR-300 has been investigated in lung cancer. Lei et al., have demonstrated that hsa-miR-300 suppression inhibited lung cancer cell proliferation, invasion, and migration [43]. Semaphorin 6D (SEMA6D) is a class VI transmembrane-type semaphoring [44]. A prior study has indicated that SEMA6D deficiency attenuated Group 2 innate lymphoid cell-induced type 2 inflammation in the lung [45]. Interestingly, the protein encoded by PPARGC1A is a transcriptional coactivator that regulates the genes involved in many diseases [46]. Shah et al., found that the PPARGC1A activator promoted mitochondrial function in lung endothelium and ameliorated lung injury in Adiponectin^−^/^−^ mice [47]. Thus, ENST00000642173 may serve as a ceRNA to downregulate hsa-miR-4291, causing the up-regulation of PPARGC1A.

Among the top 10 lncRNAs, MIR3142HG, GCC2-AS1, RAMP2-AS1, and AC145423.2 (gene symbol of ENST00000642173, NR_135290.1, NR_024462.1, ENST00000610631) have been previously reported. LncRNA MIR3142HG has been determined to mediate LPS-induced ALI in human pulmonary microvascular endothelial cells (HPMECs) by targeting the miR-450b-5p/HMGB1 axis, especially in accelerating apoptotic and inflammatory responses [48]. Another research study has further confirmed the overexpression of MIR3142HG in ALI patients, while also showing that the MIR3142HG/miR-95-5p/JAK2 axis might aggravate the progression of LPS-induced ALI in HPMEC and A549 cells [49]. These results indicated that MIR3142HG might be a potential ALI biomarker. It is worth noting that the expression of MIR3142HG in the control and LPS groups did not change significantly in BEAS-2B cells in the research studies mentioned above, which was inconsistent with the present study results. It is possible that different concentrations of LPS were responsible for this difference. Yu et al., have discovered that the expression of GCC2-AS1 was upregulated in lung adenocarcinoma tissues compared to that in normal tissue [50]. There has been an abundance of research revealing that RAMP2-AS1 has been involved in diverse biological processes, such as endothelial homeostasis [51]. The present study has screened out the top ten lncRNAs which likely play important roles in ALI, and identified the expression levels in five of them on LPS-treated BEAS-2B cells. Considering that the expression level of lncRNAs is generally low in human serum samples, lncRNA with the highest expression was chosen according to the FPKM values and the qRT-PCR results. ENST00000627824, as one of the AL139220.2 transcripts, has not been reported before and its biological function and molecular mechanism remained unexplored. The present study is the first to show its up-regulation in ALI patients compared to healthy participants, which might be a potential biomarker for ALI prediction or targeted therapy. Our study provided at least five identified lncRNAs that are worthy of further investigation for ALI biomarker prediction, as well as their underlying mechanisms and biological functions through the CNC network and the ceRNA network, a topic which also requires further exploration in vivo and in vitro.

There were some limitations in the present study. Firstly, it is inadequate to draw a reliable conclusion with a small number of blood samples, and human tissue samples are needed for further validation.- More biological experiments in vivo and in vitro are necessary to demonstrate the molecular mechanisms of important lncRNAs. The present study is therefore a small-scale exploration based on RNA-seq and bioinformatics analysis results with the intent of finding new lncRNAs that participate in the development of ALI, as well as provide a new prospective for their molecular mechanisms, especially in inflammation responses.

## 5. Conclusions

In this study, the expression profile of lncRNA and mRNA in LPS-stimulated BEAS-2B cells was revealed in order to evaluate potential biomarkers in ALI. An lncRNA–mRNA co-expression, PPI, as well as ceRNA modulatory networks, were constructed according to the RNA-seq and bioinformatics analysis results. The expressions of several important lncRNAs and mRNAs were validated by RT-qPCR in BEAS-2B cells. This is the first report confirming that ENST00000627824, one of the AL139220.2 transcripts, was significantly upregulated in ALI patients, which suggests a promising candidate for predicting ALI. All of the results provided a deeper insight into the roles of lncRNAs involved in the prediction and treatment of ALI.

## Figures and Tables

**Table 1:** The detailed information of the top 10 lncRNAs.

Transcript ID	Gene Symbol	log_2_Fold Change	Regulation	*p*-Value	Chrom	Length	Type
ENST00000576232	MRPL20-AS1	3.44576434	Up	0.0007	Chr1	585	lincRNA
ENST00000627824	AL139220.2	1.26417905	Up	0.0006	Chr1	1796	sense_intronic
ENST00000642173	MIR3142HG	3.082571424	Up	0.0043	Chr5	2204	lincRNA
NR_135290.1	GCC2-AS1	4.688605503	Up	0.0369	Chr2	569	antisense
ENST00000608576	AC073529.1	−1.970778414	Down	0.0124	ChrX	1080	lincRNA
NR_024462.1	RAMP2-AS1	−1.642451334	Down	0.0043	Chr17	1985	antisense
ENST00000649291	AL138920.1	−1.731306	Down	0.0000	Chr10	3626	lincRNA
ENST00000648279	AL161756.1	−2.383783	Down	0.0001	Chr14	2132	sense_exonic
ENST00000432142	LINC01376	−2.008642	Down	0.0272	Chr2	571	lincRNA
ENST00000610631	AC145423.2	−4.84318185	Down	0.0257	Chr12	239	sense_intronic

**Table 2:** Demographic and clinical characteristics of participants.

Parameters	Healthy (*n* = 6)	ALI/ARDS (*n* = 6)	*p*-Value
Age	50.83 ± 9.68	58.17 ± 12.19	0.28
Sex			0.99
Female	2 (33.3%)	3 (50%)	
Male	4 (66.7%)	3 (50%)	
Smoke			0.99
Yes	2 (33.3%)	2 (33.3%)	
No	4 (66.7%)	4 (66.7%)	
Hypertension			0.55
Yes	1 (16.7%)	3 (50%)	
No	5 (83.3%)	3 (50%)	

Data are presented as mean ± standard deviation (SD) or the number of cases (%).

## Data Availability

Data is available on request from the authors.

## Supplementary Materials

The following supporting information can be downloaded at: https://www.mdpi.com/article/10.3390/cimb45070389/s1, Figure S1: LncRNA and mRNA characteristics in BEAS-2B cells; Table S1: The primers of lncRNAs and mRNAs; Table S2 Differentially expressed lncRNAs; Table S3: The top 10 lncRNAs in the CNC network according to topological parameters.
