# Supplementary material for: Insight into the lncRNA–mRNA Co-Expression Profile and ceRNA Network in Lipopolysaccharide-Induced Acute Lung Injury
Source: Curr Issues Mol Biol. 2023 Jul 24;45(7):6170–89. doi: 10.3390/cimb45070389 (PMC10378513; doi:10.3390/cimb45070389)
Supplement: Supplementary file 1 [file cimb-45-00389-s001.zip › cimb-2440258-supplementary.pdf]

Supplementary Table S1 The primers of lncRNAs and mRNAs

Gene ID	Forward primer (5′-3′)	Reverse primer (5′-3′)
ENST00000627824	GAATTTGGCAGACAGGATGG	ACTCCTGTATCTTTCCCAGG
ENST00000642173	ACAGTAGCACAACGAGGAG	AGAGATGGTGCAAAGACCC
ENST00000608576	GAGACCAGATGTTTGAAGCTG	GCAATCAAGTGGCTAGTTCC
NR_024462.1	ATACCAGCTGATGTCAGTCC	CACCTTCTTCCTTCTCCCG
ENST00000432142	CTGCAGCCTCAAATTCCTG	GGGTTATTTGGAGCTGTAGTC
IL-1β	ATGATGGCTTATTACAGTGGCAA	GTCGGAGATTCGTAGCTGGA
IL-6	ACTCACCTCTTCAGAACGAATTG	CCATCTTTGGAAGGTTCAGGTTG
CXCL8	ACTGAGAGTGATTGAGAGTGGAC	AACCCTCTGCACCCAGTTTTC

Supplementary Table S2 Differentially expressed lncRNAs

lncRNA_id	FoldChange	log2FoldChange	pValue	qValue	Regulation
ENST00000316786	4.369709	2.127537	0.041437	0.999158	Up
ENST00000331787	0.031839	-4.97308	0.028905	0.999158	Down
ENST00000355869	0.071286	-3.81024	0.046764	0.999158	Down
ENST00000356906	1.886609	0.915795	0.0142	0.866453	Up
ENST00000366189	0.069264	-3.85174	1.92E-06	0.002962	Down
ENST00000366253	13.23298	3.726066	0.012049	0.832721	Up
ENST00000394864	31.79905	4.990912	0.005734	0.678873	Up
ENST00000412422	0.292954	-1.77125	0.015348	0.886575	Down
ENST00000412685	0.497342	-1.00769	0.024275	0.999158	Down
ENST00000413945	0.036525	-4.77498	0.03181	0.999158	Down
ENST00000422022	0.039306	-4.66909	0.003032	0.549611	Down
ENST00000422408	0.453294	-1.14148	0.026419	0.999158	Down
ENST00000423530	0.035752	-4.80584	0.04065	0.999158	Down
ENST00000425800	0.02717	-5.20183	0.013091	0.852565	Down
ENST00000426635	2284205	21.12326	6.53E-08	0.000263	Up
ENST00000428716	0.015827	-5.98149	0.018522	0.919714	Down
ENST00000429871	32.79592	5.035444	0.018624	0.919714	Up
ENST00000431268	1.562384	0.643749	0.02607	0.999158	Up
ENST00000432139	0.115888	-3.1092	0.037164	0.999158	Down
ENST00000432142	0.248507	-2.00864	0.027249	0.999158	Down
ENST00000432530	0.16325	-2.61484	0.036328	0.999158	Down
ENST00000432783	0.063944	-3.96705	0.033651	0.999158	Down
ENST00000434244	2.038009	1.02716	0.011469	0.823687	Up
ENST00000434872	0.011279	-6.47027	0.048812	0.999158	Down
ENST00000436286	0.413331	-1.27463	0.023739	0.999158	Down
ENST00000438107	0.04957	-4.33439	0.045819	0.999158	Down
ENST00000438412	18.53885	4.21248	0.005832	0.679104	Up

ENST00000439670	1.736276	0.795996	0.023115	0.993627	Up
ENST00000441971	0.029097	-5.10298	0.014508	0.866453	Down
ENST00000442067	59.60898	5.897458	0.033892	0.999158	Up
ENST00000442726	0.102153	-3.2912	0.000455	0.18602	Down
ENST00000443467	3.087331	1.62636	0.015294	0.886575	Up
ENST00000443947	19.83506	4.309981	0.003647	0.55619	Up
ENST00000444375	18.61323	4.218256	0.003946	0.566753	Up
ENST00000444470	2.224562	1.153521	0.009148	0.787987	Up
ENST00000444816	220.418	7.784098	0.046444	0.999158	Up
ENST00000445466	116.0851	6.859039	0.02973	0.999158	Up
ENST00000447019	4.843216	2.275965	0.036093	0.999158	Up
ENST00000447329	17.18505	4.103082	0.022249	0.985423	Up
ENST00000447413	2.428677	1.28017	0.017666	0.919714	Up
ENST00000447959	7.034468	2.814441	0.009777	0.789566	Up
ENST00000448126	0.036702	-4.768	0.035324	0.999158	Down
ENST00000451884	28.07713	4.811324	0.031061	0.999158	Up
ENST00000452057	1.689254	0.756386	0.037538	0.999158	Up
ENST00000452659	0.237116	-2.07633	0.046629	0.999158	Down
ENST00000453136	0.103803	-3.26807	0.018829	0.919714	Down
ENST00000454996	0.012251	-6.35094	0.000505	0.18602	Down
ENST00000455395	6.020022	2.589769	0.04659	0.999158	Up
ENST00000455424	0.611314	-0.71001	0.049908	0.999158	Down
ENST00000457084	0.649942	-0.62162	0.047035	0.999158	Down
ENST00000458392	1.683049	0.751077	0.047098	0.999158	Up
ENST00000474814	1.671285	0.740958	0.008653	0.778009	Up
ENST00000477192	0.333467	-1.58439	0.044935	0.999158	Down
ENST00000485071	0.566598	-0.8196	0.035835	0.999158	Down
ENST00000485898	0.336826	-1.56993	0.038245	0.999158	Down
ENST00000488315	8.181428	3.032353	0.025142	0.999158	Up
ENST00000495430	0.41418	-1.27167	0.031268	0.999158	Down
ENST00000499326	0.013134	-6.25056	0.000199	0.116859	Down
ENST00000499809	0.025345	-5.30217	0.001157	0.296785	Down
ENST00000501937	2.053963	1.03841	0.039815	0.999158	Up
ENST00000503797	0.341011	-1.55211	0.00366	0.55619	Down
ENST00000505709	0.073441	-3.76727	0.027563	0.999158	Down
ENST00000507558	28.0929	4.812134	0.035755	0.999158	Up
ENST00000508913	0.132345	-2.91763	0.004504	0.58394	Down
ENST00000509080	0.014969	-6.06184	0.002317	0.479941	Down
ENST00000513393	0.448721	-1.15611	0.025716	0.999158	Down
ENST00000514984	16.3973	4.035386	0.026644	0.999158	Up
ENST00000517747	65.15372	6.025776	0.033835	0.999158	Up

ENST00000518105	0.517314	-0.95089	0.033485	0.999158	Down
ENST00000518172	63.44514	5.987438	0.035639	0.999158	Up
ENST00000518639	1.544571	0.627207	0.044342	0.999158	Up
ENST00000522183	0.053355	-4.22824	0.02564	0.999158	Down
ENST00000523068	0.386733	-1.37059	0.023849	0.999158	Down
ENST00000523354	1.572618	0.653168	0.018065	0.919714	Up
ENST00000523643	28.5887	4.837373	0.000656	0.208568	Up
ENST00000523871	0.471215	-1.08554	0.038266	0.999158	Down
ENST00000524964	3.122694	1.642791	0.045197	0.999158	Up
ENST00000525575	0.25693	-1.96055	0.008566	0.778009	Down
ENST00000526453	2.645006	1.403271	0.0061	0.688514	Up
ENST00000526807	0.429937	-1.2178	0.021844	0.985423	Down
ENST00000527128	0.539861	-0.88934	0.045386	0.999158	Down
ENST00000532845	8.170761	3.030471	0.040294	0.999158	Up
ENST00000533615	0.030056	-5.05622	0.023968	0.999158	Down
ENST00000534671	0.013626	-6.19748	0.018209	0.919714	Down
ENST00000535076	0.411655	-1.28049	0.019929	0.937551	Down
ENST00000540904	42.61358	5.413241	0.003203	0.549611	Up
ENST00000547441	0.204169	-2.29216	0.022218	0.985423	Down
ENST00000549734	0.350117	-1.51409	0.013072	0.852565	Down
ENST00000549804	1.535487	0.618696	0.022061	0.985423	Up
ENST00000550135	0.58565	-0.77189	0.043583	0.999158	Down
ENST00000551077	4.033615	2.012073	0.048587	0.999158	Up
ENST00000552532	0.078789	-3.66586	0.013454	0.852565	Down
ENST00000553657	85.28581	6.414234	0.008078	0.778009	Up
ENST00000553829	0.477916	-1.06517	0.042313	0.999158	Down
ENST00000554036	6.115054	2.612365	0.039395	0.999158	Up
ENST00000559298	48.7774	5.608141	0.013928	0.866453	Up
ENST00000559299	0.44762	-1.15965	0.047265	0.999158	Down
ENST00000559825	0.267265	-1.90366	0.010491	0.799261	Down
ENST00000559920	0.390656	-1.35603	0.015389	0.886575	Down
ENST00000561881	0.474061	-1.07686	0.038713	0.999158	Down
ENST00000563320	0.217878	-2.19841	0.006217	0.690437	Down
ENST00000566898	26.67646	4.737495	0.030802	0.999158	Up
ENST00000567769	0.516696	-0.95261	0.012147	0.832721	Down
ENST00000568827	0.239282	-2.06322	0.019807	0.935897	Down
ENST00000568911	0.61995	-0.68978	0.00458	0.58394	Down
ENST00000569920	4.712882	2.23661	0.01338	0.852565	Up
ENST00000572358	0.502849	-0.9918	0.03784	0.999158	Down
ENST00000574741	2.283328	1.191138	0.028949	0.999158	Up
ENST00000576232	10.89628	3.445764	0.000767	0.223186	Up

ENST00000576749	0.014366	-6.12118	0.000341	0.152884	Down
ENST00000577066	3.066246	1.616474	0.002204	0.474874	Up
ENST00000579781	0.020026	-5.64198	0.004078	0.578026	Down
ENST00000582452	26.54278	4.730248	0.032052	0.999158	Up
ENST00000582549	48.62788	5.603712	0.046359	0.999158	Up
ENST00000586452	28.10148	4.812574	0.046126	0.999158	Up
ENST00000586974	0.018051	-5.79177	0.026683	0.999158	Down
ENST00000592404	46.9865	5.554174	0.007155	0.741156	Up
ENST00000593960	0.011247	-6.47434	0.018035	0.919714	Down
ENST00000594557	0.271556	-1.88068	0.033138	0.999158	Down
ENST00000594764	0.209084	-2.25785	0.029321	0.999158	Down
ENST00000596663	0.244875	-2.02988	0.037763	0.999158	Down
ENST00000597894	33.44412	5.063681	0.013429	0.852565	Up
ENST00000597946	0.083114	-3.58876	0.003306	0.549611	Down
ENST00000598139	7.463705	2.899892	0.000711	0.212827	Up
ENST00000599276	0.034608	-4.85275	0.043018	0.999158	Down
ENST00000599448	0.148532	-2.75115	0.008666	0.778009	Down
ENST00000599756	0.510485	-0.97006	0.003457	0.555782	Down
ENST00000601801	0.109263	-3.19413	9.68E-05	0.074462	Down
ENST00000602429	0.410497	-1.28456	0.01267	0.842579	Down
ENST00000602546	0.04127	-4.59875	0.000809	0.22932	Down
ENST00000602573	1.876346	0.907926	0.019007	0.920628	Up
ENST00000602718	0.240086	-2.05837	0.029167	0.999158	Down
ENST00000602802	3.432074	1.779081	0.007771	0.768055	Up
ENST00000602810	6.846941	2.77546	0.024253	0.999158	Up
ENST00000602827	0.616369	-0.69813	0.04027	0.999158	Down
ENST00000602889	2.524135	1.335789	0.046626	0.999158	Up
ENST00000604346	0.081825	-3.61131	0.000581	0.201842	Down
ENST00000604397	0.634379	-0.65658	0.006599	0.711004	Down
ENST00000604669	0.358483	-1.48002	0.027483	0.999158	Down
ENST00000605811	54.72967	5.774251	0.027394	0.999158	Up
ENST00000606243	94.71213	6.565477	0.000518	0.18602	Up
ENST00000606424	10.74831	3.426039	0.026758	0.999158	Up
ENST00000606818	0.027557	-5.18146	0.017597	0.919714	Down
ENST00000606908	2.312849	1.209671	0.009918	0.789566	Up
ENST00000607056	1.904685	0.929552	0.000239	0.116859	Up
ENST00000607861	8.66375	3.114992	0.048689	0.999158	Up
ENST00000607902	2.804564	1.487776	0.039209	0.999158	Up
ENST00000608314	0.03324	-4.91094	0.001254	0.307042	Down
ENST00000608576	0.255115	-1.97078	0.012392	0.839586	Down
ENST00000608748	38.28505	5.258709	0.00954	0.789566	Up

ENST00000608826	0.632604	-0.66063	0.014818	0.871053	Down
ENST00000609027	4.449298	2.153578	0.018867	0.919714	Up
ENST00000609218	2.85717	1.514587	0.042645	0.999158	Up
ENST00000609619	11.84962	3.566769	0.022319	0.985423	Up
ENST00000609651	0.203707	-2.29543	0.027941	0.999158	Down
ENST00000609803	2.302422	1.203152	0.014493	0.866453	Up
ENST00000610395	45.74791	5.515634	0.026111	0.999158	Up
ENST00000610631	0.034838	-4.84318	0.025772	0.999158	Down
ENST00000610658	0.038549	-4.69718	0.008288	0.778009	Down
ENST00000610681	0.361781	-1.46681	0.002013	0.451707	Down
ENST00000611056	7.315628	2.870982	0.030424	0.999158	Up
ENST00000615718	0.158156	-2.66058	0.032872	0.999158	Down
ENST00000615897	15.38797	3.943731	0.005902	0.679104	Up
ENST00000617260	0.035711	-4.8075	0.010609	0.799261	Down
ENST00000617932	0.5715	-0.80717	0.049843	0.999158	Down
ENST00000618186	0.014978	-6.06105	0.015981	0.901389	Down
ENST00000618272	0.116655	-3.09969	0.014877	0.871053	Down
ENST00000618736	0.61133	-0.70998	0.004391	0.58394	Down
ENST00000618809	24.40642	4.609189	0.028993	0.999158	Up
ENST00000619147	31.48994	4.976819	0.009216	0.787987	Up
ENST00000620465	38.73665	5.275627	4.27E-05	0.045984	Up
ENST00000621088	48.00298	5.585052	0.006135	0.688514	Up
ENST00000621354	2.052074	1.037083	0.026021	0.999158	Up
ENST00000621901	5.52565	2.466144	0.038057	0.999158	Up
ENST00000622688	134.1645	7.067859	0.022622	0.990691	Up
ENST00000622889	5.037168	2.332613	0.045148	0.999158	Up
ENST00000622921	17.59176	4.136828	0.011193	0.816987	Up
ENST00000623199	0.588845	-0.76404	0.010183	0.789566	Down
ENST00000623980	7.658051	2.936977	0.007282	0.747167	Up
ENST00000624140	0.032317	-4.95155	0.000432	0.18602	Down
ENST00000624215	0.112854	-3.14748	0.020624	0.953562	Down
ENST00000624919	0.524118	-0.93204	0.007645	0.764102	Down
ENST00000626089	0.354877	-1.49461	0.049713	0.999158	Down
ENST00000626286	0.180254	-2.4719	0.027947	0.999158	Down
ENST00000627155	0.017597	-5.8285	0.002567	0.521857	Down
ENST00000627767	0.426087	-1.23078	0.012481	0.840347	Down
ENST00000627824	2.401905	1.264179	0.000638	0.208568	Up
ENST00000628234	7.844014	2.971592	0.017856	0.919714	Up
ENST00000629474	0.02697	-5.21252	0.01846	0.919714	Down
ENST00000631832	6.115826	2.612547	0.024084	0.999158	Up
ENST00000632346	1.648084	0.72079	0.009136	0.787987	Up

ENST00000634535	0.297077	-1.75109	0.010598	0.799261	Down
ENST00000635091	0.037017	-4.75568	0.039223	0.999158	Down
ENST00000635600	0.269278	-1.89283	0.022312	0.985423	Down
ENST00000637973	0.020977	-5.57506	0.047651	0.999158	Down
ENST00000638682	0.472384	-1.08197	0.028351	0.999158	Down
ENST00000639773	3.147999	1.654435	0.035372	0.999158	Up
ENST00000641757	0.121139	-3.04526	0.016867	0.910542	Down
ENST00000642135	0.393838	-1.34433	0.035386	0.999158	Down
ENST00000642173	8.47123	3.082571	0.004316	0.58394	Up
ENST00000642872	0.532044	-0.91038	0.008489	0.778009	Down
ENST00000643135	2.867378	1.519732	0.034884	0.999158	Up
ENST00000643280	49.82364	5.638759	0.003681	0.55619	Up
ENST00000643616	12.13492	3.601093	0.020913	0.962533	Up
ENST00000643866	2.226773	1.154954	0.035732	0.999158	Up
ENST00000644040	0.303364	-1.72088	0.02617	0.999158	Down
ENST00000645023	0.468788	-1.09299	0.014261	0.866453	Down
ENST00000645656	0.406802	-1.2976	0.044991	0.999158	Down
ENST00000646488	29.14219	4.865037	0.033758	0.999158	Up
ENST00000646636	6.215326	2.63583	0.016392	0.910542	Up
ENST00000647325	0.068002	-3.87828	0.035707	0.999158	Down
ENST00000647354	1.504947	0.589713	0.010921	0.811387	Up
ENST00000647652	0.420802	-1.24879	0.041894	0.999158	Down
ENST00000648279	0.191606	-2.38378	0.000114	0.08219	Down
ENST00000648347	0.349218	-1.5178	0.043351	0.999158	Down
ENST00000649025	2.197061	1.135575	0.030001	0.999158	Up
ENST00000649087	0.257203	-1.95902	0.011096	0.816987	Down
ENST00000649291	0.301179	-1.73131	6.53E-05	0.05409	Down
ENST00000649712	0.107257	-3.22086	0.044427	0.999158	Down
ENST00000650227	0.181403	-2.46273	0.02544	0.999158	Down
ENST00000650291	0.091089	-3.45658	0.01355	0.853632	Down
ENST00000650438	11.34764	3.50432	0.03707	0.999158	Up
ENST00000650930	0.284159	-1.81523	0.032075	0.999158	Down
ENST00000651229	8.142283	3.025433	0.000667	0.208568	Up
ENST00000651519	1.561136	0.642596	0.038107	0.999158	Up
ENST00000651660	0.494324	-1.01647	0.019399	0.920628	Down
ENST00000652038	0.166301	-2.58813	0.015544	0.887144	Down
ENST00000652378	0.074835	-3.74015	0.004268	0.58394	Down
ENST00000652709	12.83915	3.682477	0.015751	0.893088	Up
ENST00000652746	0.059447	-4.07224	0.003366	0.549611	Down
NR_001568.1	0.209736	-2.25335	0.018646	0.919714	Down
NR_003697.2	1.593462	0.672165	0.045111	0.999158	Up

NR_015395.2	1.730403	0.791108	0.005389	0.659765	Up
NR_022006.1	2.514944	1.330526	0.013379	0.852565	Up
NR_023915.1	0.323932	-1.62624	0.000474	0.18602	Down
NR_024053.2	6.722711	2.749043	0.002855	0.539616	Up
NR_024236.1	0.208304	-2.26324	0.014743	0.871053	Down
NR_024284.1	109.9079	6.780151	0.011224	0.816987	Up
NR_024462.1	0.320312	-1.64245	0.004369	0.58394	Down
NR_024497.2	2.441659	1.287862	0.038197	0.999158	Up
NR_026807.2	0.494324	-1.01647	0.019399	0.920628	Down
NR_026988.1	58.84536	5.878857	0.009873	0.789566	Up
NR_027055.1	54.24859	5.761514	0.00325	0.549611	Up
NR_027159.1	0.14028	-2.83362	0.009551	0.789566	Down
NR_027295.2	0.195045	-2.35812	0.022869	0.993627	Down
NR_029376.1	0.197744	-2.33829	0.043248	0.999158	Down
NR_033319.2	0.507082	-0.97971	0.04528	0.999158	Down
NR_033827.1	2.554995	1.35332	0.034562	0.999158	Up
NR_033893.1	0.110616	-3.17637	0.009886	0.789566	Down
NR_034115.1	4.622333	2.208621	0.02593	0.999158	Up
NR_036527.1	0.281223	-1.83022	0.049974	0.999158	Down
NR_036549.1	0.460226	-1.11959	0.042604	0.999158	Down
NR_037601.1	0.6042	-0.7269	0.030925	0.999158	Down
NR_037616.1	5.441473	2.443997	0.003561	0.55619	Up
NR_038228.1	12.2195	3.611113	0.000157	0.099259	Up
NR_038276.1	0.607834	-0.71825	0.04711	0.999158	Down
NR_038281.1	0.120905	-3.04806	0.021	0.962533	Down
NR_038291.1	1.635673	0.709884	0.028605	0.999158	Up
NR_038292.1	3.611263	1.852504	6.42E-05	0.05409	Up
NR_038345.1	0.010646	-6.55351	0.015564	0.887144	Down
NR_038448.1	0.458447	-1.12517	0.008403	0.778009	Down
NR_038460.2	0.386936	-1.36983	0.034948	0.999158	Down
NR_038879.1	0.129757	-2.94611	0.001736	0.406622	Down
NR_038971.1	0.388954	-1.36233	0.00382	0.55619	Down
NR_038988.2	0.007425	-7.0733	1.60E-05	0.021543	Down
NR_039983.2	0.574191	-0.8004	0.022242	0.985423	Down
NR_040037.1	0.17079	-2.5497	0.044399	0.999158	Down
NR_040055.1	12.88143	3.687221	0.003329	0.549611	Up
NR_045021.1	0.018034	-5.79317	0.038406	0.999158	Down
NR_046089.1	0.019651	-5.66923	0.046448	0.999158	Down
NR_046542.1	0.061181	-4.03076	0.005588	0.668923	Down
NR_047532.1	4.268785	2.093825	0.025837	0.999158	Up
NR_047539.1	148.5418	7.214725	5.72E-07	0.001028	Up

NR_047540.1	0.343338	-1.5423	0.030382	0.999158	Down
NR_103456.1	0.15868	-2.65581	0.000232	0.116859	Down
NR_104014.2	0.189701	-2.3982	0.033368	0.999158	Down
NR_104183.1	0.101355	-3.30252	0.037752	0.999158	Down
NR_109832.1	0.013643	-6.1957	0.012594	0.842579	Down
NR_109909.1	11.46838	3.51959	0.025537	0.999158	Up
NR_110016.1	0.033816	-4.88615	0.03247	0.999158	Down
NR_110090.1	3.746194	1.905426	0.010456	0.799261	Up
NR_110097.1	18.86858	4.237914	0.016859	0.910542	Up
NR_110456.2	0.543979	-0.87838	0.031867	0.999158	Down
NR_110625.1	28.04646	4.809747	0.035281	0.999158	Up
NR_110688.2	0.500642	-0.99815	0.010829	0.810165	Down
NR_110725.1	0.077794	-3.68419	0.036273	0.999158	Down
NR_110787.1	0.155066	-2.68905	0.000226	0.116859	Down
NR_110917.1	0.042942	-4.54148	0.034835	0.999158	Down
NR_119377.1	0.422279	-1.24373	0.019381	0.920628	Down
NR_120363.1	0.274609	-1.86455	0.013964	0.866453	Down
NR_121614.1	2.203926	1.140076	0.00962	0.789566	Up
NR_122070.1	0.089168	-3.48733	0.040613	0.999158	Down
NR_122124.1	4.71408	2.236976	0.026591	0.999158	Up
NR_125885.1	0.02899	-5.10831	0.017539	0.919714	Down
NR_125906.1	0.29649	-1.75394	0.007001	0.739247	Down
NR_126448.1	1.907213	0.931466	0.025183	0.999158	Up
NR_130728.1	11.34601	3.504114	0.035034	0.999158	Up
NR_132346.1	0.352115	-1.50588	0.016989	0.910542	Down
NR_132748.1	8.453787	3.079598	0.018577	0.919714	Up
NR_132781.1	270.6579	8.080327	0.038703	0.999158	Up
NR_132996.1	6.466777	2.693047	0.034111	0.999158	Up
NR_134302.1	2.540847	1.34531	0.039875	0.999158	Up
NR_134594.1	39.84107	5.316184	0.020207	0.946466	Up
NR_134915.1	0.048701	-4.35991	0.043559	0.999158	Down
NR_135087.1	0.014344	-6.12345	0.003042	0.549611	Down
NR_135290.1	25.7876	4.688606	0.036908	0.999158	Up
NR_137182.1	0.055967	-4.15927	0.039099	0.999158	Down
NR_138145.1	0.247454	-2.01477	0.00147	0.351907	Down
NR_138258.1	0.018637	-5.74573	0.029919	0.999158	Down
NR_146320.1	11.31295	3.499903	0.043982	0.999158	Up
NR_146590.1	1.877557	0.908857	0.038484	0.999158	Up
NR_147505.1	0.158264	-2.65959	0.03086	0.999158	Down
NR_147886.1	0.101769	-3.29662	0.025753	0.999158	Down
NR_148945.1	1.593038	0.671781	0.023966	0.999158	Up

NR_152525.1	16.76637	4.067499	0.004522	0.58394	Up
NR_152533.1	73.71736	6.203933	0.023053	0.993627	Up
NR_152580.1	3.469306	1.794647	0.012115	0.832721	Up
NR_152738.1	0.071672	-3.80245	0.012042	0.832721	Down
NR_152798.1	4.954612	2.308772	0.003733	0.55619	Up
NR_152799.1	81.00384	6.339918	0.031395	0.999158	Up
NR_152810.1	129.1673	7.013097	4.80E-05	0.04698	Up
NR_152820.1	0.357987	-1.48202	0.0066	0.711004	Down
NR_152825.1	0.473092	-1.07981	0.035679	0.999158	Down
TCONS_00000210	1.680734	0.749091	0.046551	0.999158	Up
TCONS_00000698	0.002172	-8.84679	8.24E-08	0.000263	Down
TCONS_00000811	0.230621	-2.1164	0.04152	0.999158	Down
TCONS_00005058	3.335566	1.737932	0.018164	0.919714	Up
TCONS_00006476	2.194615	1.133968	0.019308	0.920628	Up
TCONS_00010067	3.71E-07	-21.3615	2.12E-12	2.28E-08	Down
TCONS_00013979	0.010895	-6.52017	0.009172	0.787987	Down
TCONS_00016413	0.617466	-0.69557	0.011374	0.822346	Down
TCONS_00016416	0.665955	-0.5865	0.028143	0.999158	Down
TCONS_00016420	3.328437	1.734845	3.92E-05	0.045984	Up
TCONS_00016430	0.598507	-0.74056	0.028406	0.999158	Down
TCONS_00016446	0.073499	-3.76613	0.011662	0.82653	Down
TCONS_00016454	0.599132	-0.73905	0.003141	0.549611	Down
TCONS_00017243	8.65127	3.112912	0.048698	0.999158	Up
TCONS_00022196	1880161	20.84242	9.75E-08	0.000263	Up
TCONS_00022677	1.544853	0.62747	0.010059	0.789566	Up
TCONS_00023887	1.686923	0.754394	0.014481	0.866453	Up
TCONS_00025095	2.978272	1.574475	0.016333	0.910542	Up
TCONS_00025858	0.008675	-6.84898	0.000678	0.208568	Down
XR_001737712.1	1.544688	0.627316	0.008105	0.778009	Up
XR_001738201.1	0.133278	-2.90749	0.030668	0.999158	Down
XR_001739129.1	14.55793	3.863733	0.044233	0.999158	Up
XR_001739483.1	0.365069	-1.45376	0.005007	0.62723	Down
XR_001739540.1	0.018373	-5.76627	0.006886	0.734497	Down
XR_001739646.1	2.368261	1.243828	0.014558	0.866453	Up
XR_001740466.2	4.98228	2.316806	0.000854	0.23588	Up
XR_001740875.2	0.153618	-2.70258	0.033392	0.999158	Down
XR_001741431.1	2.146215	1.101795	0.022911	0.993627	Up
XR_001741555.1	0.006356	-7.2976	4.88E-07	0.001028	Down
XR_001741723.1	33.39344	5.061493	0.023195	0.993627	Up
XR_001744266.2	0.040666	-4.62003	0.00273	0.539616	Down
XR_001745175.1	1.712416	0.776033	0.030803	0.999158	Up

XR_001745395.1	0.038926	-4.68312	0.017103	0.91215	Down
XR_001746149.2	4.66864	2.223002	0.002777	0.539616	Up
XR_001746732.2	4.065681	2.023497	0.044979	0.999158	Up
XR_001746959.2	0.057494	-4.12045	0.044535	0.999158	Down
XR_001747329.1	0.09694	-3.36676	0.037093	0.999158	Down
XR_001747440.1	6.157139	2.62226	0.025453	0.999158	Up
XR_001747598.1	6.37864	2.673249	0.014042	0.866453	Up
XR_001748100.1	0.258558	-1.95144	0.044021	0.999158	Down
XR_001749012.1	0.589107	-0.7634	0.025357	0.999158	Down
XR_001749031.1	3.614537	1.853811	0.00102	0.274589	Up
XR_001749100.1	0.252335	-1.98659	0.037197	0.999158	Down
XR_001749102.1	0.247361	-2.01531	0.041317	0.999158	Down
XR_001749149.1	11.74908	3.554476	0.034173	0.999158	Up
XR_001749294.1	0.200985	-2.31484	0.049928	0.999158	Down
XR_001749298.1	0.259173	-1.94801	0.01643	0.910542	Down
XR_001749299.1	5.202306	2.379151	0.046923	0.999158	Up
XR_001749785.1	13.8022	3.786826	0.024567	0.999158	Up
XR_001750974.1	44.41802	5.473073	0.045102	0.999158	Up
XR_001751642.1	14.47176	3.855168	0.019107	0.920628	Up
XR_001752318.2	0.640779	-0.6421	0.039627	0.999158	Down
XR_001752718.2	0.021012	-5.57263	0.038094	0.999158	Down
XR_001753092.2	2.593631	1.374973	0.037086	0.999158	Up
XR_001754469.2	0.015426	-6.01848	0.018828	0.919714	Down
XR_001754566.1	16.42799	4.038084	0.013331	0.852565	Up
XR_001754953.1	0.036463	-4.77742	0.014144	0.866453	Down
XR_001755026.2	0.091993	-3.44233	0.004607	0.58394	Down
XR_001755487.1	29.80694	4.897576	0.025294	0.999158	Up
XR_001755788.1	99.68193	6.63926	0.000266	0.124404	Up
XR_001755960.2	0.350967	-1.51059	0.033816	0.999158	Down
XR_002956216.1	0.231684	-2.10977	0.03625	0.999158	Down
XR_002956362.1	69.88523	6.126916	0.001804	0.413546	Up
XR_002956368.1	1.633217	0.707717	0.044381	0.999158	Up
XR_002956527.1	0.064755	-3.94886	0.003783	0.55619	Down
XR_002956578.1	22.07967	4.464647	0.020588	0.953562	Up
XR_002956729.1	0.37012	-1.43393	0.000506	0.18602	Down
XR_002957586.1	0.260853	-1.93869	0.012213	0.832721	Down
XR_002957604.1	0.07382	-3.75985	0.034642	0.999158	Down
XR_002957710.1	3.004741	1.587241	0.017458	0.919714	Up
XR_002957877.1	0.021528	-5.53762	0.047828	0.999158	Down
XR_002958195.1	0.056807	-4.13779	0.007646	0.764102	Down
XR_002958423.1	0.018478	-5.75808	0.000234	0.116859	Down

XR_002958445.1	7.668458	2.938937	0.001198	0.300072	Up
XR_002958529.1	0.105204	-3.24874	0.029475	0.999158	Down
XR_002958570.1	0.090227	-3.47029	0.047743	0.999158	Down
XR_002959441.1	0.008552	-6.86946	0.003367	0.549611	Down
XR_002959446.1	64.10546	6.002375	0.018213	0.919714	Up
XR_427154.4	32.84494	5.037599	0.021673	0.98518	Up
XR_429965.3	26.47751	4.726695	0.007068	0.739247	Up
XR_923257.3	0.376591	-1.40893	0.044953	0.999158	Down
XR_926349.2	40.38721	5.335826	0.016701	0.910542	Up
XR_926624.3	0.058848	-4.08685	0.005926	0.679104	Down
XR_927172.3	0.167392	-2.5787	0.009829	0.789566	Down
XR_927952.3	0.353725	-1.4993	0.00912	0.787987	Down
XR_929618.3	0.171805	-2.54115	0.002131	0.468537	Down
XR_930134.2	0.01753	-5.83406	0.005507	0.66664	Down
XR_930176.2	0.036158	-4.78952	0.045074	0.999158	Down
XR_930276.3	0.010709	-6.54497	0.008641	0.778009	Down
XR_930803.2	0.101251	-3.304	0.046018	0.999158	Down
XR_931667.2	0.168653	-2.56787	0.035433	0.999158	Down
XR_933035.3	25.37613	4.6654	0.048298	0.999158	Up
XR_933690.2	0.019863	-5.65376	0.008289	0.778009	Down
XR_936802.2	36.4726	5.188741	0.011591	0.82653	Up
XR_939886.2	6.221562	2.637277	0.021086	0.962533	Up
XR_939896.2	7.396597	2.886862	0.025533	0.999158	Up
XR_940258.2	0.110722	-3.17498	0.048347	0.999158	Down
XR_940325.2	0.162198	-2.62417	0.008131	0.778009	Down
XR_940570.2	0.109109	-3.19615	0.002844	0.539616	Down
XR_940788.3	0.016247	-5.94364	0.006308	0.693412	Down
XR_940911.2	27.44774	4.778616	0.046906	0.999158	Up
XR_945302.2	4.778646	2.256602	0.00766	0.764102	Up
XR_945304.2	0.02432	-5.3617	0.00109	0.286381	Down
XR_947755.2	11.97777	3.582287	0.023243	0.993627	Up
XR_947991.2	2.884109	1.528126	0.000151	0.099259	Up
XR_948815.2	0.101949	-3.29408	0.010187	0.789566	Down
XR_949653.3	0.508184	-0.97658	0.010045	0.789566	Down
XR_950154.2	0.407014	-1.29685	0.031992	0.999158	Down
XR_950234.3	0.396888	-1.3332	0.002294	0.479941	Down
XR_950387.3	0.498021	-1.00572	0.038959	0.999158	Down

Supplementary Table S3 The top 10 lncRNAs in the CNC network according to topological parameters

Transcript ID	Degree	EPC	Closeness	Radiality
ENST00000576232	134	259.161	362.33333	4.09593
ENST00000642173	133	267.175	362.91667	4.10894
ENST00000627824	133	258.476	361.66667	4.09268
ENST00000608576	130	255.776	359.66667	4.08293
NR_135290.1	130	257.625	360.91667	4.09919
NR_024462.1	125	252.698	355.08333	4.05041
ENST00000649291	124	253.22	356.66667	4.07642
ENST00000648279	121	251.916	351.91667	4.03089
ENST00000432142	119	248.62	350.08333	4.01789
ENST00000610631	118	242.234	350.91667	4.03415

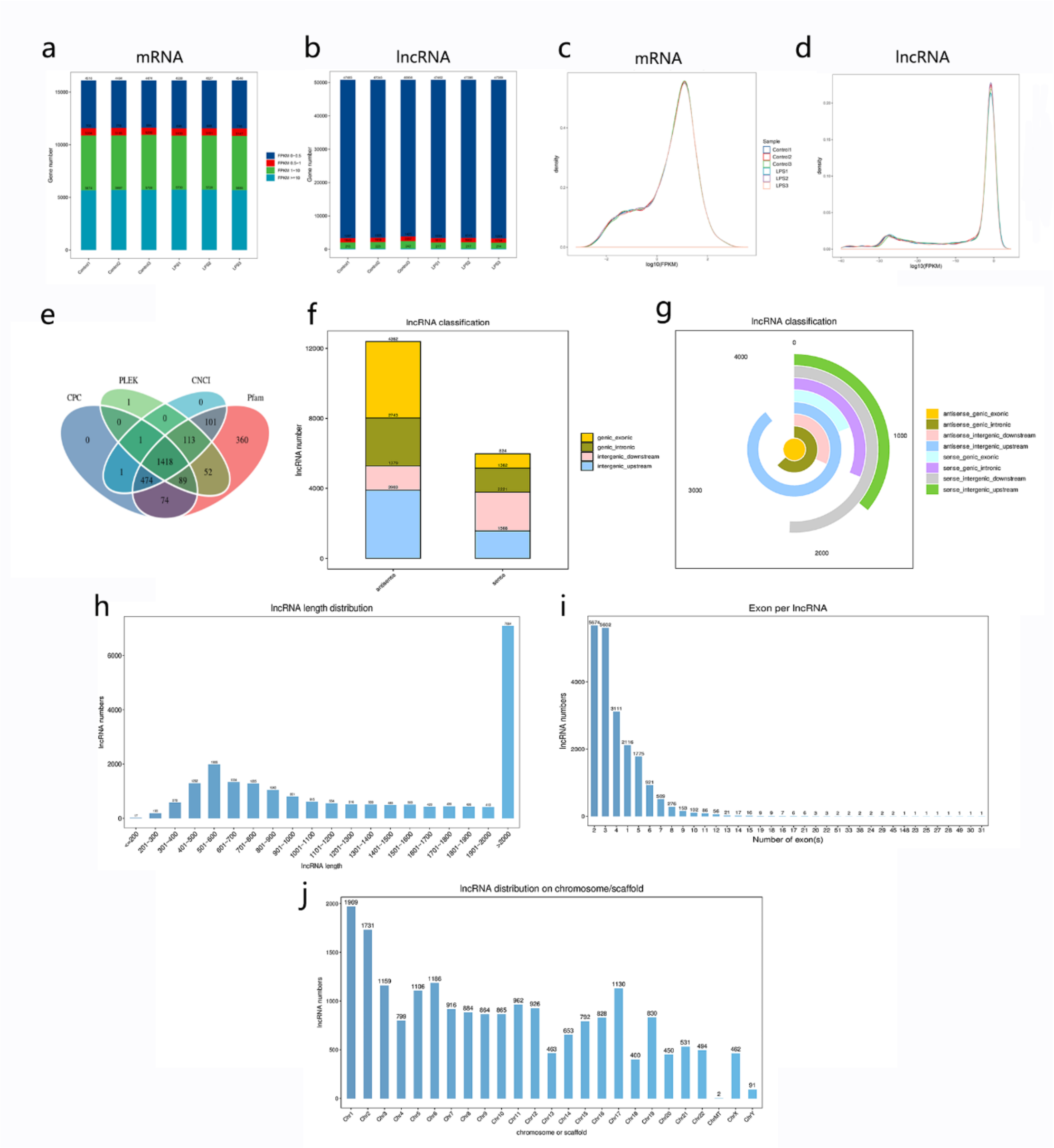

Supplementary Figure S1 LncRNA and mRNA characteristics in BEAS-2B cells. (**a,b**) FPKM values distribution of mRNA and lncRNA. (**c,d**) Gene expression density of mRNA and lncRNA. (**e**) Prediction of novel lncRNAs by 4 algorithms. **(f,g**) LncRNA classification. (**h**) LncNRA length distribution. (**i**) Number of exons for lncRNA. (**j**) LncRNA distribution on chromosomes.
